# Prediction of urine culture results by automated urinalysis with digital flow morphology analysis

**DOI:** 10.1038/s41598-021-85404-1

**Published:** 2021-03-16

**Authors:** Dokyun Kim, Seoung Chul Oh, Changseung Liu, Yoonjung Kim, Yongjung Park, Seok Hoon Jeong

**Affiliations:** 1 Department of Laboratory Medicine, Gangnam Severance Hospital, Yonsei University College of Medicine, 211 Eonju-ro Gangnam-gu, Seoul, 06273 South Korea; 2 Research Institute of Bacterial Resistance, Yonsei University College of Medicine, Seoul, South Korea; 3 Department of Laboratory Medicine, School of Medicine, Kangwon National University, Chuncheon, South Korea

**Keywords:** Diagnostic markers, Predictive markers, Bacterial infection, Urinary tract infection

## Abstract

To investigate the association between the results of urinalysis and those of concurrent urine cultures, and to construct a prediction model for the results of urine culture. A total of 42,713 patients were included in this study. Patients were divided into two independent groups including training and test datasets. A novel prediction algorithm, designated the UTOPIA value, was constructed with the training dataset, based on an association between the results of urinalysis and those of concurrent urine culture. The diagnostic performance of the UTOPIA value was validated with the test dataset. Six variables were selected for the equation of the UTOPIA value: age of higher UTI risk [odds ratio (OR), 2.069125], female (OR, 1.400648), nitrite (per 1 grade; OR, 3.765457), leukocyte esterase (per 1 grade; OR, 1.701586), the number of WBCs (per 1 × 10^6^/L; OR, 1.000121), and the number of bacteria (per 1 × 10^6^/L; OR, 1.004195). The UTOPIA value exhibited an area under the curve value of 0.837 when validated with the independent test dataset. The UTOPIA value displayed good diagnostic performance for predicting urine culture results, which would help to reduce unnecessary culture. Different cutoffs can be used according to the clinical indication.

## Introduction

Urinary tract infection (UTI) is the most common bacterial infection acquired in the community and in healthcare facilities. The prevalence of UTI is estimated to be 11% of the overall population, and almost half of adult women suffer from UTI at least once in their lifetime^1,2^. Clinical manifestations of UTI are mostly mild; however, the disease could develop serious complications, especially in certain high-risk populations including infants, pregnant women, and aged population^3^. Therefore, early diagnosis and empirical antimicrobial treatment is essential to improve clinical outcomes of patients with UTI^4^.

The gold standard for definitive diagnosis of UTI is detection of the pathogen by bacterial culture of a urine specimen^5^, and an antimicrobial susceptibility profile can be obtained by testing clinical isolates. However, urine culture is a time-consuming procedure, and the microbial spectrum of causative organisms in UTIs is narrow. Therefore, routine cultures are often not necessary to manage patients with uncomplicated UTIs, and only urinalysis either by test strip analysis and/or sediment analysis are recommended for the decision of patient management^6^. Among the components of test strip analysis, leukocyte esterase (LE) and nitrite are commonly used to diagnose UTI in routine clinical practices. Urine LE positive indicates pyuria, and urine nitrite positive indicates the presence of nitrate-reducing bacteria. However, diagnostic performance of these tests is not sufficiently high to be used alone due to limitations of the test principle^7^.

Test strip analysis is traditionally done by the dipstick based on physicochemical reactions, and the results are interpreted using a reflectometer. Automated urinalysis systems including sample preparation, aliquot, and reading have been introduced to improve test throughput and efficiency and to reduce labor and time. In addition, microscopic examination of urine sediment is also widely used to diagnose urinary tract diseases by identifying various types of cells, casts, and crystals in a urine sample. However, manual microscopic examination is a time-consuming procedure and requires expertise to maintain consistency of the result interpretation. Recently, different types of automated urine sediment analysis systems have been introduced. Among them, the iQ200 (Beckman Coulter Inc., Brea, CA, US) is an automated digital imaging-based system that uses flow morphology analysis to classify particles in a urine sample based on multiple parameters including size, shape, contrast, and texture. This instrument has exhibited satisfactory analytical performance for the quantitation of red blood cells (RBCs), white blood cells (WBCs), and epithelial cells compared with other automated sediment analysis systems and manual microscopic methods^8^.

Here, we evaluated an association between the results of urinalysis obtained by the iRICELL system including the iQ200 automated urine sediment analysis instrument with results of concurrent urine cultures. We also aimed to construct a simple but practical prediction model for the positive urine culture with the results of urinalysis including automated urine sediment analysis.

## Results

### Patient characteristics and urine culture results

The median (1st–3rd quartiles) age of the 42,713 patients was 56 (24–69), and 38.7% (n = 16,519) of the patients were included in the high-risk age group (Table 1). Almost half (50.7%, n = 21,635) of the subjects were male, and two thirds (70.3%, n = 30,036) of the subjects were hospitalized patients. The median (1st–3rd quartiles) difference in reception time between urinalysis and urine culture was 0.3 (0.1–19.1) minutes, and the median difference in report time was 39.8 (23.7–62.7) hours. The results of urine culture were positive for 17.1% (n = 7292) of the patients, and 89.2% (n = 6506) of these were positive with a single pathogen, 4.5% (n = 325) with a single pathogen and a possible pathogen below the threshold, 3.3% (n = 220) with a single pathogen and a single normal flora, and 3.0% (n = 220) with two pathogens. The most common pathogen isolated in this study was *Escherichia coli* (54.9%, n = 4121 among 7512) followed by *Enterococcus faecalis* (11.7%, n = 878), *Klebsiella pneumoniae* (6.5%, n = 491), and *Enterococcus faecium* (5.3%, n = 400) (Supplementary Table 1). Patients in the urine culture-positive group exhibited a significantly higher proportion of high-risk age group (53.3% vs 35.7%, *P* < 0.0001) and lower proportion of males (33.8% vs 54.1%, *P* < 0.0001) than the urine culture-no growth or contamination group (Table 1 and Supplementary Table 2).

**Table 1:** Patient characteristics and results of urinalysis according to the urine culture results.

Variable^a^	Urine culture-no growth or contamination	Urine culture-positive	*P*-value	Total
	35,421 (82.9%)	7292 (17.1%)		42,713 (100.0%)
Age (year)	55 (24–69)	58 (19–74)	< 0.0001	56 (24–70)
Age group with high risk for UTI	12,636 (35.7%)	3883 (53.3%)	< 0.0001	16,519 (38.7%)
Male	19,171 (54.1%)	2464 (33.8%)	< 0.0001	21,635 (50.7%)
Hospitalization	24,901 (70.3%)	5135 (70.4%)	0.8388	30,036 (70.3%)
Difference in reception time between urinalysis and urine culture (minute)	0.3 (0.1–18.2)	0.3 (0.1–22.9)	0.5109	0.3 (0.1–19.1)
Time to report results of urinalysis (minute)	23.0 (16.0–33.7)	23.5 (16.3–34.7)	< 0.0017	23.1 (16.1–33.8)
Difference in report time between urinalysis and urine culture (hour)	36.8 (22.8–56.5)	60.6 (45.5–82.4)	< 0.0001	39.8 (23.7–62.7)
Time to report results of urine culture (hour)	36.7 (23.0–56.2)	60.6 (45.6–82.5)	< 0.0001	39.8 (23.9–62.6)
**Test strip analysis**				
Specific gravity	1.015 (1.010–1.021)	1.013 (1.008–1.018)	< 0.0001	1.015 (1.009–1.021)
pH	6.0 (5.0–6.5)	6.0 (5.5–7.0)	< 0.0001	6.0 (5.0–6.5)
Protein	Trace (Negative–Trace)	Trace (Negative–1 +)	< 0.0001	Trace (Negative–1 +)
Glucose	Negative (Negative–Negative)	Negative (Negative–Negative)	0.1894	Negative (Negative–Negative)
Blood/red blood cell	Negative (Negative–Trace)	Trace (Negative–1 +)	< 0.0001	Negative (Negative–Trace)
Nitrite	Negative (Negative–Negative)	Negative (Negative–1 +)	< 0.0001	Negative (Negative–Negative)
Leukocyte esterase	Negative (Negative–Negative)	2 + (Negative–3 +)	< 0.0001	Negative (Negative–1 +)
**Digital flow morphology analysis (× 10**^**6**^**/L)**				
Red blood cell	5 (2–14)	12 (3–53)	< 0.0001	5 (2–18)
White blood cell	6 (2–16)	76 (9–573)	< 0.0001	7 (3–26)
Epithelial cell	1 (0–3)	2 (0–7)	< 0.0001	1 (0–4)
Cast	0 (0–0)	0 (0–0)	< 0.0001	0 (0–0)
Bacteria	0 (0–1)	2 (0–13)	< 0.0001	0 (0–1)
Urine culture results	No growth, 23,454 (66.2%)	Single pathogen, 6506 (89.2%)		
	Possible contamination, 8260 (23.3%)	Single pathogen with possible pathogen below the threshold, 325 (4.5%)		
	Single possible pathogen below the threshold, 1894 (5.3%)	Single pathogen with single normal flora, 241 (3.3%)		
	Single normal flora, 1,259 (3.6%)	Two pathogens, 220 (3.0%)		
	Miscellaneous, 554 (1.6%)			

*UTI* urinary tract infection.

^a^Categorical variables and continuous variables are presented by number (%) and median (1st–3rd quartiles), respectively.

**Table 2:** The results of multivariate analysis by logistic regression for the prediction of positive urine culture with 42,713 patients.

Factor	Odds ratio	(95% confidence interval)	*P *value
Age of higher risk	1.967	(1.848–2.095)	< 0.0001
Female	1.483	(1.389–1.584)	< 0.0001
Hospitalized patient	1.174	(1.096–1.259)	< 0.0001
**Test strip analysis**			
SG (per 0.001 increase)	0.979	(1.025–1.098)	< 0.0001
pH (per 1.0 increase)	1.061	(1.025–1.098)	0.0007
Protein (per 1 grade increase)	0.979	(0.918–1.043)	0.5127
Glucose (per 1 grade increase)	1.021	(0.984–1.06)	0.2675
Blood (per 1 grade increase)	0.935	(0.894–0.978)	0.0035
Nitrite (per 1 grade increase)	3.952	(3.679–4.246)	< 0.0001
Leukocyte esterase (per 1 grade increase)	1.736	(1.691–1.782)	< 0.0001
**Digital flow morphology analysis**			
RBC (per 1 × 10^6^/L increase)	1.000	(1.000–1.000)^a^	< 0.0001
WBC (per 1 × 10^6^/L increase)	1.000	(1.000–1.000)^b^	< 0.0001
Epithelial cell (per 1 × 10^6^/L increase)	0.996	(0.995–0.998)	< 0.0001
Cast (per 1 × 10^6^/L increase)	1.001	(0.994–1.008)	0.7592
Bacteria (per 1 × 10^6^/L increase)	1.006	(1.005–1.007)	< 0.0001

^a^1.000028 (1.000015–1.000041).

^b^1.000115 (1.000086–1.000145).

### The results of urinalysis according to the culture results

The results of test strip and sediment analyses according to the urine culture results are summarized in Table 1. Except for urine glucose, all parameters were significantly different between the two groups. By the multivariate binary logistic regression, three patient factors including high-risk age [odds ratio (OR), 1.967], female (OR, 1.483), and hospitalization (OR, 1.174) were significantly associated with positive results of the urine culture (*P* < 0.0001 for each) (Table 2). Among the test strip results, pH (OR, 1.061 per 1.0 increase; *P* = 0.0007), nitrite (OR, 3.952 per 1 grade increase; *P* < 0.0001), and LE (OR, 1.736 per 1 grade increase; *P* < 0.0001) were independent risk factors for positive urine culture. Among the parameters of automated sediment analysis, the numbers of RBCs (OR, 1.000 per 1 × 10^6^/L increase), WBCs (OR, 1.000 per 1 × 10^6^/L increase), epithelial cells (OR, 1.001 per 1 × 10^6^/L increase), and bacteria (OR, 1.006 per 1 × 10^6^/L increase) showed significant associations with positive results of the urine culture (*P* < 0.0001 for each).

Among the variables exhibiting significant associations with positive urine culture, six variables including age of higher risk (OR, 2.069125), female (OR, 1.400648), nitrite (OR, 3.765457 per 1 grade increase), LE (OR, 1.701586 per 1 grade increase), the number of WBCs (OR, 1.000121 per 1 × 10^6^/L increase), and the number of bacteria (OR, 1.004195 per 1 × 10^6^/L increase) were selected considering the effect size of each variable by multivariable binary logistic regression in the training dataset with 21,522 patients (*P* < 0.0001 for all variables; Supplementary Table 3). An equation to predict urine culture results was constructed with the constant and coefficients of independently significant variables as follows:

$${\text{UTOPIA value }} = { }\frac{1}{{1 + e^{{\left( {2.803456{ } - { }0.727126x1{ } - { }0.336935x2{ } - { }1.325869x3{ } - { }0.531561x4{ } - { }0.000121x5{ } - { }0.004186x6} \right)}} }} \times 100$$

where,

- *x*_1_ = 1, if a **patient is at high-risk age (≤ 1 or ≥ 70 years old), otherwise *x*_1_ = 0
- *x*_2_ = 1 for female; *x*_2_ = 0 for male
- *x*_3_ = grade of nitrite by test strip analysis (0.5 when the result is trace or weak positive)
- *x*_4_ = grade of LE by test strip analysis (0.5 when the result is trace or weak positive)
- *x*_5_ = number of WBCs by digital flow morphology analysis (1 × 10^6^/L)
- *x*_6_ = number of bacteria by digital flow morphology analysis (1 × 10^6^/L).

**Table 3:** Possible cutoffs and the utility of the UTOPIA value.

Cutoff for UTOPIA value	Youden's index	Diagnostic performance (95% CI) for the prediction of urine culture positive results in the test dataset				% Estimated culture reduction^a^/% false negative^b^ at a given culture positive prevalence of				
		Sensitivity	Specificity	PPV^c^	NPV^c^	5.0%	10.0%	15.8%^c^	20.0%	25.0%
> 5.72	0.204	0.972 (0.966–0.977)	0.232 (0.226–0.238)	0.192 (0.191–0.194)	0.978 (0.973–0.982)	22.2/0.6	21.1/1.3	20.0/2.2	19.1/2.9	18.1/3.8
> 6.54	0.279	0.950 (0.942–0.956)	0.330 (0.323–0.337)	0.210 (0.208–0.212)	0.972 (0.968–0.976)	31.6/0.8	30.2/1.7	28.6/2.8	27.4/3.7	26.0/4.9
> 7.86	0.394	0.904 (0.893–0.913)	0.490 (0.482–0.497)	0.250 (0.246–0.253)	0.964 (0.961–0.968)	47.0/1.0	45.0/2.1	42.7/3.6	41.1/4.7	39.1/6.1
> 15.11	0.522	0.661 (0.644–0.676)	0.862 (0.857–0.867)	0.473 (0.462–0.484)	0.931 (0.928–0.934)	83.6/2.0	81.0/4.2	77.9/6.9	75.7/9.0	73.1/11.6
> 23.03	0.497	0.596 (0.579–0.612)	0.901 (0.897–0.905)	0.531 (0.518–0.544)	0.922 (0.919–0.925)	87.6/2.3	85.1/4.7	82.3/7.8	80.2/10.1	77.7/13.0
> 34.21	0.448	0.498 (0.481–0.515)	0.950 (0.947–0.953)	0.651 (0.635–0.668)	0.910 (0.907–0.912)	92.8/2.7	90.5/5.6	87.9/9.0	86.0/11.7	83.8/15.0
> 92.61	0.115	0.118 (0.107–0.129)	0.998 (0.997–0.998)	0.900 (0.868–0.924)	0.857 (0.856–0.859)	99.2/4.5	98.6/8.9	97.9/14.3	97.5/18.1	96.9/22.8

*CI* confidence interval, *PPV* positive predictive value, *NPV* negative predictive value.

^a^Assuming that the UTOPIA values are determined to be negative based on a given cutoff value and thus subsequent urine cultures are not carried out.

^b^Proportion of cases with urine culture positive results among subjects showing negative by the UTOPIA value, i.e., 1—NPV at a given cutoff and culture positive prevalence.

^c^The prevalence of positive urine culture in the test dataset was 15.8%.

### Diagnostic performance of the UTOPIA value

**Figure 1:**
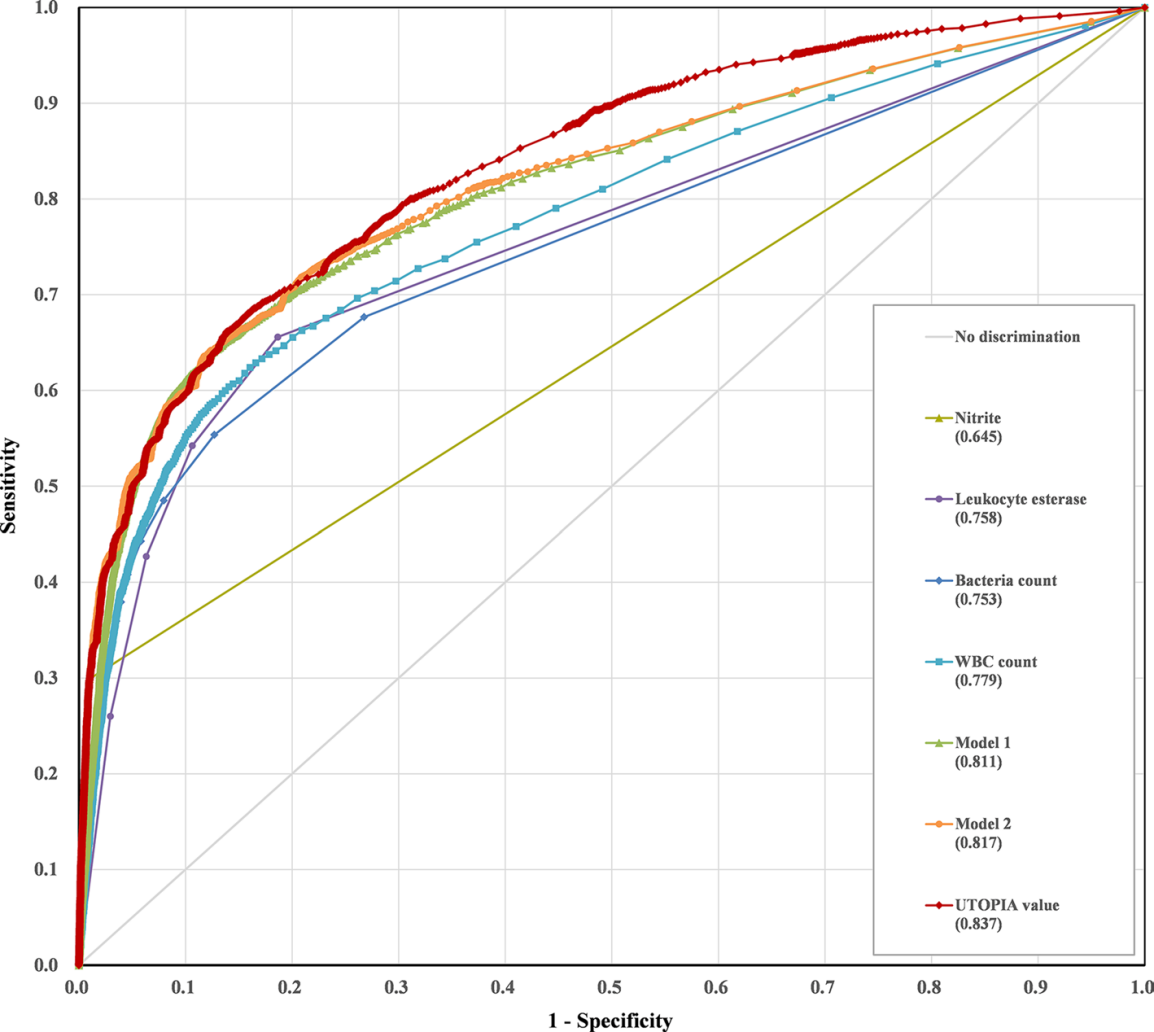
Receiver operating characteristics (ROC) curve analysis of the urinalysis in the prediction of urine culture positive results in the test dataset. The area under the curve (AUC) of the model 2 (combination of nitrite, leukocyte esterase, and WBC and bacteria counts) was higher than that of the model 1 (combination of WBC and bacteria counts) (*P* = 0.0002), and the UTOPIA value showed the highest AUC value among those of other tests (*P* < 0.0001).

To validate the diagnostic performance of the UTOPIA value for predicting results of urine culture, ROC curves were constructed with the independent test dataset composed of 21,191 patients from different periods, and the AUC of the UTOPIA value was 0.837 (95% CI = 0.829–0.845), which is significantly higher than that of nitrite (AUC = 0.645; 95% CI = 0.637–0.653), LE (AUC = 0.758; 95% CI = 0.749–0.767), the number of bacteria (AUC = 0.753; 95% CI = 0.743–0.762), and the number of WBCs (AUC = 0.779; 95% CI = 0.769–0.789) (*P* < 0.0001 for all comparison, Fig. 1). In addition, the UTOPIA value also exhibited higher AUC value than the other models including the Model 1 (AUC = 0.811; 95% CI = 0.802–0.820) which consisted of WBCs and bacteria counts by automated sediment analysis, and the Model 2 (AUC = 0.817; 95% CI = 0.808–0.826) which was composed with LE, nitrite, and the variables of Model 1 (Fig. 1).

When using > 15.11 as a cutoff for the UTOPIA value, which showed the highest Youden’s index, the sensitivity, specificity, positive predictive value (PPV), and negative predictive value (NPV) were 0.661, 0.862, 0.473, and 0.931, respectively (Table 3). A cutoff value of > 6.54 exhibited sensitivity of 0.950 and specificity of 0.330, while a cutoff value of > 34.21 showed specificity of 0.950 and sensitivity of 0.498.

## Discussion

Microscopic examination of urine particle is a useful tool for diagnosing UTI, although the gold standard for diagnosis is urine culture. To date, three different types of automated urine sediment analyzers have been introduced. Sysmex UF-1000i (Sysmex Corporation, Kobe, Japan) utilizes the flow cytometric method. This analyzer measures numbers of cells, bacteria, and casts by electrical impedance per flow sample volume, sizes the components by forward light-scatter, and nuclear and cytoplasmic characteristics using fluorescent dye^9^. Another instrument the cobas u701 (Roche Diagnostics International, Rotkreuz, Switzerland), which was first introduced as UriSed (77 Elektronika, Budapest, Hungary)^10^, takes 15 microscopic images per urine sample prepared in cuvettes that mimic glass slides used in manual microscopic examination, and the result images are analyzed by particle recognition software^11^. The iQ200 investigated in this study is an automated digital imaging-based system that uses flow morphology analysis. In previous studies, the iQ200 system showed reliable performance in counting RBCs, WBCs, and epithelial cells in terms of imprecision and linearity and showed good correlation with manual microscopic sediment analysis and other automated analyzers^8,12,13^.

There have been several studies to evaluate possible associations between the results of microscopic urine sediment examinations and those of urine culture. A meta-analysis for predicting positive urine culture by the results from the Sysmex UF-1000i or UF-100 systems showed good sensitivity, using the number of WBCs (pooled sensitivity, 0.87) and bacteria (pooled sensitivity, 0.92) counted by flow cytometry as indicators^14^. The number of bacteria in urine specimens obtained by Accuri C6 (BD Biosciences, San Jose, CA, US) showed good correlation with the results of urine culture when a cutoff value for urine culture positive was ≥ 10^5^ CFU/mL^15^. A recent interlaboratory study exhibited that the absence of microorganisms in the iQ200 screen was the strongest solitary predictor for a negative culture result with a sensitivity of 90.5%, and higher sensitivity (95.2%) could be obtained by the algorithm based on the presence of microorganisms and the number of WBCs^16^. Another study with the iQ200 system exhibited an acceptable NPV of 97.7% and approximately 50% reduction of urine culture when using WBC ≥ 4/HPF as a cutoff in predicting urine culture results, but the PPV was only 24.5% in the same study^17^. The scoring system suggested by Foudraine et al., which was composed of clinical symptoms including dysuria and urgency and the number of WBCs obtained by the iQ200 analyzer, gave good diagnostic performance with a high AUC value of 0.950 for predicting positive blood cultures^18^. However, the diagnostic performance of a test could vary according to the characteristics and composition of cases and controls included in each study, thus it would be difficult to directly compare diagnostic performance among the studies. In addition, the definition of significant growth in urine culture in each study was different, thus it is also difficult to generalize the diagnostic performance of a test in the literature.

The UTOPIA value was designed to predict the positive urine culture with the variables including demographic conditions including age of higher risk for UTI and sex, results of urinalysis including nitrite, LE, and the numbers of WBCs and bacteria, and was validated with an independent dataset consisting of 21,191 patients in a different time period from the subjects in the training dataset. The distribution of the prevalence of UTI along with age was a J-shape with a higher frequency among the very young and a gradual increase with age, and the prevalence was significantly higher for women than men, as previously described^19^. By simply adding these two risk factors as variables of the prediction algorithm, the UTOPIA value exhibited better diagnostic performance than the other models those are consisted of only the variables from urinalysis (Fig. 1). This work provides a novel approach to predict the result of urine culture with the patients’ risk factors and the results of urinalysis. In addition, the UTOPIA value was designed with easy-to use data in order to incorporate into a laboratory information system easily, and thus can be automatically calculated immediately after urinalysis.

When validated with the independent test dataset, the UTOPIA value provided a good AUC value of 0.837 in the prediction of positive urine culture with high NPVs regardless of applied cutoffs. With the prevalence of our dataset (15.8%), the NPV was 0.978 (95% CI = 0.973–0.982) when applying a cutoff for the UTOPIA value of > 5.72, and 20.0% of total culture cases was estimated to be reduced at the expense of 2.2% of false negative results, i.e. 1—NPV based on the UTOPIA value (Table 3). Since the prevalence of the urine culture positive results can vary depending on factors such as the country, region, and patient age, appropriate cutoffs for the UTOPIA value would need to be applied for each clinical laboratory. The cost of urine culture according to the countries would be also considered. Using different cutoffs according to the allowable false negatives in each laboratory, the UTOPIA value would be utilized to reduce unnecessary urine cultures. Meanwhile, the utility of the UTOPIA value would be low if it is used for determining whether to start early empirical antibiotic treatment before the culture results are reported. In this instance, PPV of the UTOPIA value was 0.900 even when applying a high cutoff of > 92.61. Consequently, it can be applied to only 2.1% of the total patients because there would be only small number of patients showing positive results by the UTOPIA value with that high cutoff, and there would be false positive cases of 10.0%, i.e. 1—PPV, among the 2.1% of total patients as well.

In our data, the proportion of urine culture contamination cases was 28.0%, and they included in the control group to make a practical and accurate model for predicting the results of urine culture in actual clinical microbiology laboratories. In addition, the contamination group exhibited intermediate characteristics when comparing with urine culture negative and positive groups (Supplementary Table 2). If contamination cases were excluded from the regression model, the 1diagnostic performance of UTOPIA value would be over-estimated.

One limitation of our study is that it was performed with the retrospective design, and 19.1% of total cases were excluded due to inaccurate quantitative results obtained by iQ200. Therefore, possible selection bias would be considered when interpreting our results. However, a large number of patients was included to minimize unpredictable bias and to enhance the statistical power with narrow CIs for the results in this study, and the study population was divided into two independent datasets including training and test datasets to improve the reliability and external validity of our results. Despite this effort, the validation of diagnostic performance of the UTOPIA value in a single hospital would be another limitation of this study, even though the independent dataset from a different time period was used in the validation. Multicenter evaluation for the diagnostic performance of the UTOPIA value calculated by the equation in this study would be helpful in the generalized application of the UTOPIA value. Additionally, we investigated the results from a single type of test strip analyzer and flow morphology analyzer among several automated urinalysis systems each utilizing different test principles and showing different semi-quantitative results for chemical parameters including LE. Separate prediction algorithms according to the type of urinalysis systems could also be developed by applying a similar approach to our study.

In conclusion, we designed a novel prediction algorithm for urine culture results based on the results of urine test strip analysis and digital flow morphology analysis, namely the UTOPIA value. The UTOPIA value showed good diagnostic performance with possibility of reducing unnecessary urine culture and flexibility to apply different cutoff values. This prediction algorithm can be used to predict urine culture results 1 to 3 days before the culture results are reported, and also has the advantage of being easily incorporated electronically into a laboratory information system. Further evaluation on the usefulness of the UTOPIA value in various clinical settings should be considered.

## Materials and methods

### Study design and patients

**Figure 2:**
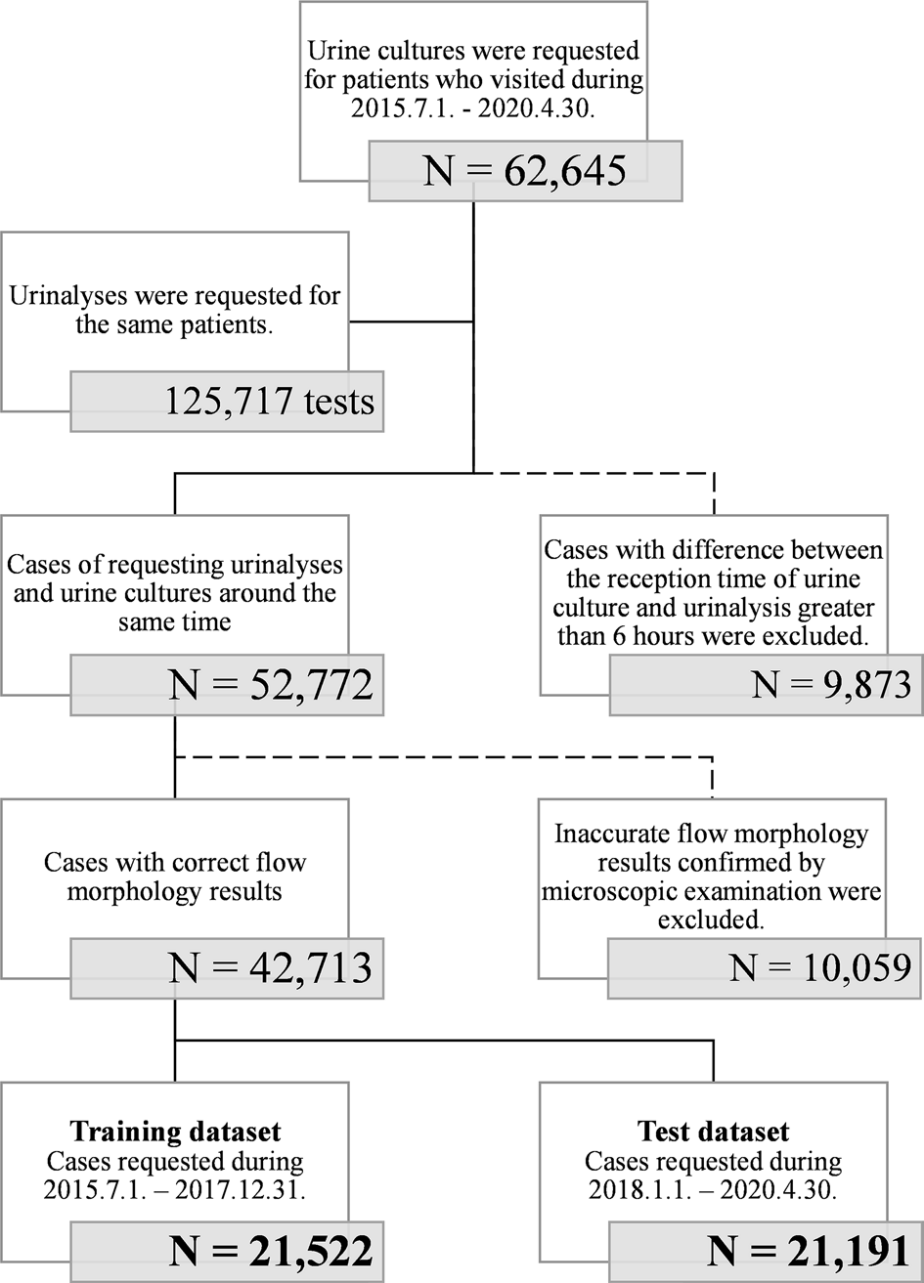
Study design and classification of cases. Solid lines indicate cases included in the analysis, while dotted lines represent excluded subjects.

From July 2015 to April 2020, a total of 62,656 patients were subjected to urine cultures for suspected UTIs in a tertiary hospital in South Korea. Among them, 52,772 patients were subjected to urinalyses within 6 h before or after urine culture, and 10,059 patients were excluded due to incomplete or inaccurate automated urine sediment analysis results. Finally, 42,713 patients were enrolled in this study (Fig. 2). Patients included in this study were divided into two datasets by the time of receipt: (1) a training dataset with 21,522 patients: cases requested between July 2015 and December 2017, and (2) a test dataset with 21,191 patients: cases requested between January 2018 and April 2020. This retrospective cross-sectional case–control study, designated the UTOPIA study (Urinalysis-based Timely and On-the-spot Prediction of Infection Algorithm), was designed to develop a simple and useful algorithm to predict urine culture results using results of urinalysis. Patient characteristics including demographic information and type of admission were investigated by reviewing electronic medical records. The protocol of this study was approved by the Institutional Review Board of Gangnam Severance Hospital (Approval No. 3-2020-0169), and the requirement of an informed consent of the participants was waived by the IRB. All methods used in this study were also performed in accordance with the relevant guidelines and regulations.

### Urine culture

The results of urine culture were retrieved from the electronic medical records. Urine culture was performed according to the standard protocol of the local microbiology laboratory. Briefly, one microliter of urine sample was inoculated on MacConkey agar and Blood agar, and the number of colonies was counted after an 18-h incubation to calculate bacterial load. Bacterial identification was performed using a Matrix-Assisted Laser Desorption Ionization Time-of-Flight Mass Spectrometer (MALDI-TOF MS). To make an accurate prediction model for positive urine culture, the results of urine cultures were categorized into “Positive” and “No growth or contamination”.

### Automated urinalysis with digital flow morphology analysis

The results of test strip analysis and sediment analysis by digital flow morphology analysis were retrieved from the electronic medical records. Automated urinalysis were performed using the iRICELL system (Beckman Coulter Inc., Brea, CA), which consisted of the iChem VELOCITY urine chemistry analyzer and the iQ200 SPRINT urine microscopy analyzer, following the manufacturer’s instructions. For the iQ200 instrument, approximately 1.3 mL of urine passes through a flow cell, and a digital camera captures 500 images of magnified sample. Then, the Auto-particle Recognition (APR) software (current version 7.1.4) interprets the captured images. The flow morphology interpretation with flags for suspicious errors or abnormal results by the APR software were reviewed with on-screen images by operators. Based on comprehensive consideration with on-screen images, previous urinalysis results of the same patient, and the test strip results concurrently obtained by iChem, cases with discrepant interpretations between operators and the analyzing software were subjected to manual microscopic sediment examination. If needed, the results for these cases were corrected as the number of cells per high-power field by manual microscopic examination, and were excluded from our study due to inaccurate quantitative values for RBCs, WBCs, and epithelial cells by the iQ200 analyzer in those cases. During the study period, three quality control materials for the iChem VELOCITY including IRISpec CA, CB, and CC (Beckman Coulter Inc.) and two materials for the iQ200 including iQ positive and negative controls (Beckman Coulter Inc.) were run every eight hours.

### Definition

The high-risk age group for UTI was defined as patients younger than 2 years or older than 69 years considering high positive rates of urine culture according to national surveillance study^20^ and positive rates of urine culture according to age in our data. A positive urine culture was determined when a single uropathogen (bacterial load ≥ 10,000 CFU/mL) or two uropathogens (bacterial load of each species ≥ 100,000 CFU/mL) were recovered. Uropathogens include Gram-negative bacilli, *Staphylococcus aureus*, *Candida* species, *Enterococcus* species, and *Aerococcus urinae*, as previously described^21^. Cases with more than three species recovered from urine culture were considered as contamination regardless of the quantity of bacterial growth^21^.

### Statistical analysis

All statistical analyses were performed by Analyse-it for Microsoft Excel Method Evaluation Edition version 5.65.3 (Analyse-it Software, Ltd., Leeds, UK) and IBM SPSS Statistics 25 (IBM Corp., Armonk, NY, US). Patient characteristics and the results of urinalysis according to the groups classified by the urine culture results were compared with chi-square tests for categorical variables and Mann–Whitney U tests for continuous variables. Binary logistic regression with the results of urine culture as the dependent variable and those of urinalysis and patients’ characteristics as the multivariate independent variables was performed to determine the coefficient for each independent variable in the regression model. With the regression model equation, the UTOPIA value for each case in the test dataset was calculated to predict the probability for positive urine culture, and diagnostic performance of the UTOPIA value for the prediction of urine culture results was evaluated by calculating the area under the curve (AUC) value. All statistical analyses in this study were considered significant when the *P* value was < 0.05.

## Supplementary Information

Supplementary Tables.

## References

[1] Medina M, Castillo-Pino E (2019). An introduction to the epidemiology and burden of urinary tract infections. Ther. Adv. Urol..

[2] Chu CM, Lowder JL (2018). Diagnosis and treatment of urinary tract infections across age groups. Am. J. Obstet. Gynecol..

[3] Flores-Mireles AL, Walker JN, Caparon M, Hultgren SJ (2015). Urinary tract infections: epidemiology, mechanisms of infection and treatment options. Nat. Rev. Microbiol..

[4] Lee SS, Kim Y, Chung DR (2011). Impact of discordant empirical therapy on outcome of community-acquired bacteremic acute pyelonephritis. J. Infect..

[5] Kang CI (2018). Clinical practice guidelines for the antibiotic treatment of community-acquired urinary tract infections. Infect. Chemother..

[6] Meyrier, A. Sampling and evaluation of voided urine in the diagnosis of urinary tract infection in adults. in *UpToDate* (ed. Post, T. W.) (2019).

[7] Demilie T, Beyene G, Melaku S, Tsegaye W (2014). Diagnostic accuracy of rapid urine dipstick test to predict urinary tract infection among pregnant women in Felege Hiwot Referral Hospital, Bahir Dar, North West Ethiopia. BMC Res. Notes.

[8] Linko S (2006). Analytical performance of the Iris iQ200 automated urine microscopy analyzer. Clin. Chim. Acta Int. J. Clin. Chem..

[9] Ben-Ezra J, Bork L, McPherson RA (1998). Evaluation of the Sysmex UF-100 automated urinalysis analyzer. Clin. Chem..

[10] Zaman Z (2010). Urine sediment analysis: Analytical and diagnostic performance of sediMAX—a new automated microscopy image-based urine sediment analyser. Clin. Chim. Acta Int. J. Clin. Chem..

[11] Wesarachkitti B (2016). Performance evaluation and comparison of the fully automated urinalysis analyzers UX-2000 and Cobas 6500. Lab. Med..

[12] Wah DT, Wises PK, Butch AW (2005). Analytic performance of the iQ200 automated urine microscopy analyzer and comparison with manual counts using Fuchs-Rosenthal cell chambers. Am. J. Clin. Pathol..

[13] Lamchiagdhase P (2005). Urine sediment examination: a comparison between the manual method and the iQ200 automated urine microscopy analyzer. Clin. Chim. Acta Int. J. Clin. Chem..

[14] Shang YJ (2013). Systematic review and meta-analysis of flow cytometry in urinary tract infection screening. Clin. Chim. Acta Int. J. Clin. Chem..

[15] Moshaver B (2016). Fast and accurate prediction of positive and negative urine cultures by flow cytometry. BMC Infect. Dis..

[16] Russcher A (2016). Interlaboratory collaboration for optimized screening for urinary tract infection. J. Clin. Microbiol..

[17] Lee JM, Baek DJ, Park KG, Han E, Park YJ (2019). Clinical usefulness of iQ200/iChem Velocity workstation for screening of urine culture. BMC Infect. Dis..

[18] Foudraine DE (2018). Use of automated urine microscopy analysis in clinical diagnosis of urinary tract infection: defining an optimal diagnostic score in an academic medical center population. J. Clin. Microbiol..

[19] Foxman B (2010). The epidemiology of urinary tract infection. Nat. Rev. Urol..

[20] Lee H (2018). Antimicrobial resistance of major clinical pathogens in South Korea, May 2016 to April 2017: first one-year report from Kor-GLASS. Euro surveillance : bulletin Europeen sur les maladies transmissibles Eur. Commun. Dis. Bull..

[21] Leber AL (2016). Clinical Microbiology Procedures Handbook.

