# Supplementary material for: Prediction of urine culture results by automated urinalysis with digital flow morphology analysis
Source: Sci Rep. 2021 Mar 16;11:6033. doi: 10.1038/s41598-021-85404-1 (PMC7966378; doi:10.1038/s41598-021-85404-1)
Supplement: Supplementary file 1 — Supplementary Tables. [file 41598_2021_85404_MOESM1_ESM.pdf]

Prediction of Urine Culture Results by Automated Urinalysis with Digital Flow Morphology Analysis

Dokyun Kim[1,2], Seoung Chul Oh[1], Changseung Liu[1,2,3], Yoonjung Kim[1], Yongjung Park[1#], Seok Hoon Jeong[1,2]

[1]Department of Laboratory Medicine, Gangnam Severance Hospital, Yonsei University College of Medicine, Seoul, South Korea

[2]Research Institute of Bacterial Resistance, Yonsei University College of Medicine, Seoul, South Korea

[3]Department of Laboratory Medicine, School of Medicine, Kangwon National University, Chuncheon, South Korea

Supplementary Table 1. Pathogens isolated from urine cultures of 7,292 patients

Classification	Pathogen	Number of isolates (%)	
Gram-negative bacilli	*Escherichia coli*	4,121	(54.9)
	Klebsiella pneumoniae	491	(6.5)
	Klebsiella oxytoca	100	(1.3)
	Other *Klebsiella* species	16	(0.2)
	Enterobacter aerogenes	134	(1.8)
	Enterobacter cloacae	85	(1.1)
	Enterobacter asburiae	13	(0.2)
	Other *Enterobacter* species	3	(0.0)
	Pseudomonas aeruginosa	213	(2.8)
	Other *Pseudomonas* species	9	(0.1)
	Proteus mirabilis	138	(1.8)
	Proteus vulgaris	15	(0.2)
	Other *Proteus* species	2	(0.0)
	Citrobacter freundii	72	(1.0)
	Citrobacter koseri	49	(0.7)
	Other *Citrobacter* species	8	(0.1)
	Acinetobacter baumannii	23	(0.3)
	Acinetobacter pittii	12	(0.2)
	Acinetobacter junii	8	(0.1)
	Other *Acinetobacter* species	18	(0.2)
	Morganella morganii	52	(0.7)
	Serratia marcescens	47	(0.6)
	Stenotrophomonas maltophilia	8	(0.1)
	Providencia species	7	(0.1)
	Other gram-negative bacilli	18	(0.2)
Gram-positive cocci	*Enterococcus faecalis*	878	(11.7)
	Enterococcus faecium	400	(5.3)
	Other *Enterococcus* species	24	(0.2)
	Streptococcus agalactiae	208	(2.8)
	Other *Streptococcus* species	4	(0.1)
	Staphylococcus aureus	130	(1.7)
	Aerococcus urinae	1	(0.0)
Yeast	*Candida albicans*	99	(1.3)
	Candida glabrata	53	(0.7)
	Candida tropicalis	35	(0.5)
	Candida parapsilosis	8	(0.1)
	Other *Candida* species	10	(0.1)
Total		7,512	(100.0)

Supplementary Table 2. Patients' characteristics and the results of urinalysis stratified according to the urine culture results

Variables	Groups by urine culture results			*P*-value
	Negative (n = 23,454)	Contamination (n = 11,967)	Positive (n = 7,292)	
No. of patients with high risk age	7,536 (32.1%)	5,100 (42.6%)	3,883 (53.3%)	<0.0001
No. of female patients	9,006 (38.4%)	7,244 (60.5%)	4,828 (66.2%)	<0.0001
Nitrite	Negative (Negative to Negative)	Negative (Negative to Negative)	Negative (Negative to 1+)	<0.0001
Leukocyte esterase	Negative (Negative to Negative)	Negative (Negative to 1+)	2+ (Negative to 3+)	<0.0001
WBC count ($\times 10^6$/L)	5 (2 to 14)	8 (3 to 24)	76 (9 to 573)	<0.0001
Bacteria count ($\times 10^6$/L)	0 (0 to 0)	0 (0 to 1)	2 (0 to 13)	<0.0001

Data are shown as no. (%) or median (1st to 3rd quartiles).

Supplementary Table 3. The results of multivariate binary logistic regression with selected factors as independent variables and urine culture results as the outcome variable for constructing a prediction model using the training dataset consisting of 21,522 patients

Factor	Odds ratio	(95% confidence interval)	*P*-value
Age of higher risk	2.069125	(1.903922–2.248663)	<0.0001
Female	1.400648	(1.284317–1.527517)	<0.0001
Urinalysis			
Nitrite (per 1 grade increase)	3.765457	(3.427472–4.136771)	<0.0001
Leukocyte esterase (per 1 grade increase)	1.701586	(1.646313–1.758714)	<0.0001
Flow morphology analysis WBC (per 1 $\times 10^6$/L increase)	1.000121	(1.000085–1.000157)	<0.0001
Bacteria (per 1 $\times 10^6$/L increase)	1.004195	(1.003042–1.005349)	<0.0001
